# Fluorescence Sensing of Caffeine in Tea Beverages with 3,5-diaminobenzoic Acid

**DOI:** 10.3390/s20030819

**Published:** 2020-02-03

**Authors:** Chenxu Du, Chaoqun Ma, Jiao Gu, Lei Li, Guoqing Chen

**Affiliations:** 1 School of Science, Jiangnan University, Wuxi 214122, China; 6171203006@stu.jiangnan.edu.cn (C.D.); machaoqun@jiangnan.edu.cn (C.M.); jiaogu@jiangnan.edu.cn (J.G.); lli@jiangnan.edu.cn (L.L.); 2 Jiangsu Provincial Research Center of Light Industrial Optoelectronic Engineering and Technology, Wuxi 214122, China

**Keywords:** 3,5-diaminobenzoic acid, fluorescence probe, molecular electrostatic potential, caffeine

## Abstract

A rapid, selective and sensitive method for the detection of caffeine in tea infusion and tea beverages are proposed by using 3,5-diaminobenzoic acid as a fluorescent probe. The 3,5-diaminobenzoic acid emits strong fluorescence around 410 nm under the excitation of light at 280 nm. Both the molecular electrostatic potential analysis and fluorescent lifetime measurement proved that the existence of caffeine can quench the fluorescence of 3,5-diaminobenzoic acid. Under the optimal experimental parameters, the 3,5-diaminobenzoic acid was used as a fluorescent probe to detect the caffeine aqueous solution. There exists a good linear relationship between the fluorescence quenching of the fluorescent probe and the concentration of caffeine in the range of 0.1–100 μM, with recovery within 96.0 to 106.2%, while the limit of detection of caffeine is 0.03 μM. This method shows a high selectivity for caffeine. The caffeine content in different tea infusions and tea beverages has been determined and compared with the results from HPLC measurement.

## 1. Introduction

It is well known that caffeine is found in more than 60 plants such as coffee, cocoa beans, tea leaves, which are easy to aggregate with natural polyphenols [1,2,3]. It is responsible for the particular flavor of caffeine-containing beverages [4]. Caffeine is a central nervous stimulant, which can call a person’s strength back. Therefore, caffeine-containing tea and tea beverages are very popular in the world. However, too much caffeine is bad for a person’s body. The concentration of caffeine in the body is an important indicator for health. The consumption of caffeine is regarded to be harmless for adults (<100 mg/day). When consumption of caffeine is more than 200 mg/day, it could lead to coma and death [5]. Excessive consumption of caffeine could cause some diseases, such as headaches, heart disease, carcinogenesis and kidney malfunction [6]. Therefore, it is very important to control the content of caffeine in products. It is essential to select a method with high selectivity, sensitivity and short analysis time to determine caffeine content.

A number of methods have been used to determine caffeine in samples of different teas and tea beverages, including high-performance liquid chromatography (HPLC) [7], gas chromatography-mass spectrometry (GC-MS) [8], high performance liquid chromatography-mass spectrometry (HPLC-MS) [9], electrophoresis [10], capillary electrophoresis (CE) [11], high performance thin layer chromatography (HPTLC) [12], UV-visible spectrophotometry [13], voltammetry [14], micellar liquid chromatography [15] and ion mobility spectrometry [16]. However, these techniques are often time-consuming, laborious and costly. In addition, these methods may require some pre-processing, which will increase the possibility of error in the experiment. Compared to these techniques, fluorescence analysis has the characteristic advantage of high sensitivity, and it has become an effective method in the field of analysis [17,18,19,20,21,22,23,24].

Caffeine does not exhibit fluorescent properties but Amit K. Ghosh et al. successfully detected caffeine in different teas with Acridine Orange (AO) as a probe [25]. Sebastien Rochat et al. described the fluorescence method for the detection of caffeine [26]. Jordan Smith et al. have demonstrated a fluorescence approach for the rapid, simple and selective detection of caffeine in real tea beverages using aspirin as probe [27]. Wang et al. reported the first BODIPY-based fluorescence turn-on caffeine sensor working in aqueous solution, which provided the ideas and basis for my subsequent experiments [28].

In this study, we have used 3,5-diaminobenzoic acid for the fluorescence determination of caffeine in tea infusion and tea beverages. Caffeine itself does not have fluorescence properties, while 3,5-diaminobenzoic acid has strong fluorescent properties. In the experiment, we discovered the noncovalent interaction between caffeine and 3,5-diaminobenzoic acid, which resulted in the fluorescence quenching of 3,5-diaminobenzoic acid. Based on the fluorescence quenching effect of caffeine on 3, 5-diaminobenzoic acid, a simple, effective, low-cost fluorescent probe was designed. The optimum conditions for detecting caffeine were determined by fluorescence experiments. According to the interference experiments, the excellent selectivity of 3,5-diaminobenzoic acid to caffeine was proved. Finally, we successfully determined the caffeine content in several actual samples and compared them with the value obtained with the HPLC method. The limit of detection of caffeine was 0.03 μM. This is a new method for detecting caffeine content in tea infusion and tea beverages by fluorescence method.

## 2. Materials and Methods

### 2.1. Chemicals and Materials

Caffeine, catechin, epicatechin (ECG), tea polyphenol, L-Theanine and flavonol were procured from Beijing Putian Tongchuang Biotechnology Co., Ltd. Tea polysaccharide was procured from Shanghai Yuanye Biotechnology Co., Ltd. 3,5-diaminobenzoic acid was procured from Sinopharm Chemical Reagent Co., Ltd. The purity of Tea polysaccharide is greater than 50% and the purity of other Chemicals is 92% or higher. Fuxi green tea (sample S1), Bama black tea (sample S2), Chunchashe green tea drink (sample S3), Dongfangshuye green tea drink (sample S4), Dongfangshuye black tea drink (sample S5), and Dongfangshuye oolong tea drink (sample S6) were obtained from the local market for the analysis of caffeine content. Ultrapure water was obtained from the Labonova purification system and all the experiments were carried out with freshly prepared solutions.

### 2.2. Apparatus

All fluorescence studies in this paper were performed using the FLS920 type steady-state and time-resolved fluorescence spectrometers. All HPLC tests are performed on the Agilent 1200. The time-resolved spectra are recorded using a time correlated single photon counting (TCSPC) with a 273.7 nm light-emitting diode laser. The electrostatic potential distribution is simulated by Guassian 09.

### 2.3. Experimental Conditions

The concentration of 3,5-diaminobenzoic acid solution is 4 × 10^−5^ M, the temperature is 25 ℃, and the pH is 6. Under the optimum conditions, 0.5 mL, 3,5-diaminobenzoic acid aqueous solution was added to different concentration of caffeine (2 ml) aqueous solutions. Three minutes later, the fluorescence spectra of the mixed solutions were measured at an excitation wavelength of 280 nm and the fluorescence intensity was recorded at the emission wavelength of 410 nm. The slit widths were 5.0 and 5.0 nm for excitation and emission, respectively. All experiments were performed for three times, and the average of those values is reported.

### 2.4. Preparation of Samples

Quantitative determinations of caffeine were carried out using commercially sold samples. In the case of tea samples, 0.5 g of the tea leaf sample was added into 50 mL of boiling water in a beaker. Then the mixture was allowed to stand after stirring for one minute. After cooling to room temperature, the supernatant was taken out and was centrifuged at 10,000 rpm for 10 minutes to remove the sediments from the solution. In order to get clear solutions, the solutions were filtered using a 0.22 μm membrane. In the case of tea beverages samples, tea beverage solutions were degassed for 15 minutes using an ultrasonic bath, and then the method above was repeated. Finally, the solutions were filtered with a 0.22 μm membrane.

### 2.5. Component Influence

To verify the high selectivity of 3,5-diaminobenzoic acid for caffeine in actual tea samples, solutions of flavonoids, catechin, epicatechin, tea polyphenols, chlorophyll, caffeine were prepared at a concentration of 100 μM. In the component interference experiment, a 0.5 ml solution of 3,5-diaminobenzoic acid (4 × 10^−5^ M) was mixed with 2 mL above solutions (100 μM) under optimum conditions. The fluorescence intensity of solutions was determined at an excitation wavelength of 280 nm and an emission wavelength of 410 nm.

### 2.6. High-Performance Liquid Chromatography

Six tea samples and a caffeine sample were taken and filtered through a 0.22 μm microporous membrane for HPLC analysis in a liquid phase bottle. HPLC conditions were as follows: injection volume, 5 μL; column, Zorbax SB-C18 (4.6 mm × 150 mm); mobile phase, acetonitrile/water/TFA 10/90/0.05; gradient elution, 50/50/0.05; determination wavelength, 280 nm; flow rate, 0.8 mL/min; column temperature, 30 ℃.

## 3. Results

### 3.1. The Reason for Fluorescence Quenching

**Figure 1:**
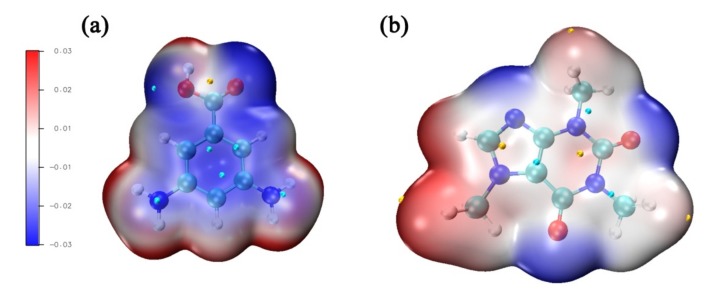
(**a**). The molecular electrostatic potential of 3,5-diaminobenzoic acid. (**b**). The molecular electrostatic potential of caffeine.

The electrostatic potential of surface molecules is used to explain their interactions between caffeine and 3,5-diaminobenzoic acid. In Figure 1, we can distinguish the charged regions of the molecule by color scale, which allows us to understand the interactions between molecules. The blue regions represent the negative potential and the red regions are related to the positivity potential of the molecule. As shown in Figure 1a,b, the potential near the benzene ring in the 3,5-diaminobenzoic acid is negative, and the potential near the carbon atom in caffeine is positive, demonstrating that these two molecules form caffeine-3,5-diaminobenzoic acid complex more easily. Moreover, we also found that the potential near the oxygen atom in 3,5-diaminobenzoic acid is negative, indicating that this atom is more likely to capture free protons, while the potential near the hydrogen atom in caffeine is positive, which means that the combination of the two is very easy. In a pure aqueous system, the complex forms due to π-stacking between caffeine and 3,5-diaminobenzoic acid. Fluorescence lifetime measurements can be used to verify our assumption that, because of the formation of the complex, the fluorescence lifetime will change. From Figure S1, it could be seen that 3,5-diaminobenzoic acid and caffeine with 3,5-diaminobenzoic acid have different fluorescence lifetimes. This phenomenon indicates the fluorescence quenching of the 3,5-diaminobenzoic due to the formation of the complex.

### 3.2. Determination of Optimal Conditions

In this study, the concentration of 3,5-diaminobenzoic acid is one of the important factors in fluorescence quenching efficiency. The results of caffeine detection are greatly affected by the concentration of the probe. Therefore, it is very important to select an appropriate concentration of probe. As seen in Figure S2a, the concentration of 3,5-diaminobenzoic acid is in the range 10–100 μM and the concentration of caffeine remains at 50 μM (*F_0_* represents the fluorescence intensity before adding the caffeine and *F* stands for the fluorescence intensity after adding the caffeine). The percentage of fluorescence quenching changes quickly with the different concentration of 3,5-diaminobenzoic acid. The fluorescence intensity of mixed solution increases rapidly when the concentration of 3,5-diaminobenzoic acid is in the range 30–70 μM. Figure S2a shows a good linear range and high sensitivity when the concentration of 3,5-diaminobenzoic acid was 40 μM. According to the results, concentration of 3,5-diaminobenzoic acid was determined to be 40 μM for further studies.

As we can see from Figure S2b, the fluorescence quenching is also heavily affected by the pH of the solution. When the pH value was less than 5, the hydroxyl and amino groups of 3,5-diaminobenzoic acid were protonated. Under alkaline conditions, the fluorescence quenching becomes weaker, as hydroxyl ionization produces anions. To further verify the optimal pH value of the experiment, we performed fluorescence response experiments of pH with 3,5-diaminobenzoic acid solution. As shown in Figure S3, the fluorescence intensity of the 3,5-diaminobenzoic acid decreases quickly when the pH value is in the range ~4.5–7.5. The change in fluorescence intensity can be fitted with the Boltzmann equation

$$I=1,977,840+1,679,400/(1+\exp(\mathrm{pH}-5.75)/0.61)(R^{2}=0.996,pKa=5.75)$$

Hence, a pH value of six was chosen for further studies. In addition, the change in temperature had an important effect on the experiment. Evidently, the fluorescence-quenching intensity remains almost constant in the temperature range of 10–50 °C from Figure S4a. Therefore, the temperature of 25 ℃ was the optimal temperature for the experiment. Finally, there was a need to examine the fluorescence response time. As shown in Figure S4b, the changes in fluorescence intensity were recorded from 1 to 20 minutes after adding caffeine. The fluorescence intensity starts to drop sharply within 3 minutes after the addition of caffeine and the fluorescence intensity keeps constant for the next 17 minutes. This indicates that the reaction of 3,5-diaminobenzoic acid with caffeine is completed within 3 minutes. Therefore, all subsequent experiments were performed after three minutes.

### 3.3. Fluorescence Quenching Experiments of 3,5-Diaminobenzoic Acid

**Figure 2:**
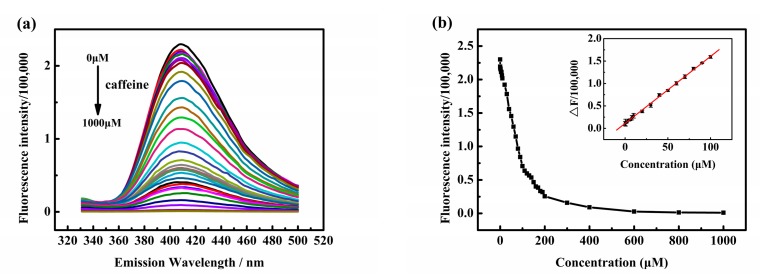
(**a**) Fluorescence changes in 3,5-diaminobenzoic acid with different concentrations of caffeine (0–1000 μM), excitation at 280 nm. (**b**) Fluorescence intensity of 3,5-diaminobenzoic acid with addition of different concentrations of caffeine. The corresponding inset is the fluorescence intensity linear graph of 3,5-diaminobenzoic acid with different concentrations of caffeine (0.1 μM–100 μM).

Next, under the optimal conditions, fluorescence-quenching experiments of 3, 5-diaminobenzoic acid and caffeine at different concentrations were performed by the standard addition method. As is portrayed in Figure 2a, the fluorescence intensity of the mixed solution was decreased rapidly with the increase in caffeine concentration in the range of 0-100 μM. When the concentration of caffeine is more than 100 μM, the fluorescence intensity of the mixed solution decreases slowly. According to this phenomenon, we determined that the optimal concentration of caffeine is in the range of 0.1 μM–100 μM. As we can see from Figure 2b, the change can be fitted with a straight line equation: $Y\left(∆F\right)=1494x+11156$; here, *x* is the concentration of caffeine and the correlation coefficient squared (R^2^) is 0.9991, $∆F=F_{0}-F$ represents the fluorescence-quenching intensity, and *F_0_* and *F* are the fluorescence intensity before and after adding the caffeine. The linear equation can be used as a standard equation for the subsequent determination of caffeine in actual samples. However, the detection limit was 0.03 μM from 3 *σ*/*K* with *K* the slope of the fitting line and σ the standard deviation of blank measurements. The result of this work was compared with other methods in Table 1. This shows that our method gives a lower detection limit and a good linear range compared with most other methods, which proves the superiority of our method.

### 3.4. Selectivity

**Figure 3:**
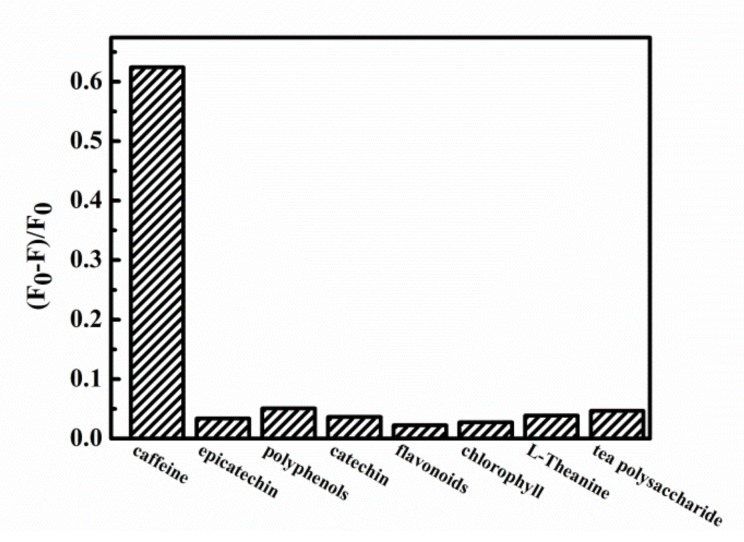
Fluorescence response of the 3,5-diaminobenzoic acid on caffeine and other major components in tea.

To further demonstrate the reliability of the fluorescence sensor, the fluorescence response of the 3,5-diaminobenzoic acid was studied on other major components in tea. The main components in tea are L-Theanine, tea polysaccharide, flavonoids, catechin, epicatechin, tea polyphenols, chlorophyll, caffeine. Therefore, these components were selected as interfering substances. All experiments were evaluated under the same conditions. The concentrations of all the interfering substances were fixed at 100 μM, because the average content of these interfering substances (after dilution) in tea infusion and tea beverages are in the range of 0.1–150 μM. Tea polysaccharide is a compound polysaccharide, so the concentration of tea polysaccharide was fixed at 10 μg/mL. As we can see from Figure 3, under the same experimental conditions, other interfering substances have little effect on the fluorescence quenching of 3,5-diaminobenzoic acid. The quenching intensity of caffeine for 3,5-diaminobenzoic acid is much higher than other interfering substances, which proves its excellent selectivity to caffeine. According to the result, this method can be used to determine caffeine in actual samples.

### 3.5. Analysis in Actual Samples

In order to verify the accuracy of the experimental method, the recovery experiment was carried out with six actual samples. At first, the concentration of samples should be diluted into the linear range of our experiment. Then, 5 μM of caffeine standard solution was added to the samples and the fluorescence intensity of the solution was recorded. At last, the amount of caffeine and recovery were determined by standard addition method. All experimental data are averaged after three experiments. As shown in Table 2, the results of recovery were calculated in the range 96.0%–106.2%. This proves the accuracy of our experimental method.

**Figure 4:**
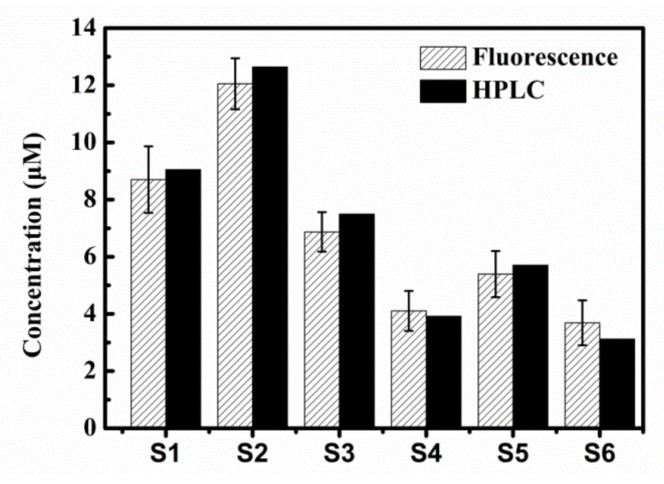
Content of caffeine in six samples was determined by HPLC (black) or by fluorescence spectroscopy (shadow).

Finally, HPLC testing was performed to determine caffeine in six actual samples (Figure S5–11). We obtained the amount of caffeine in the actual sample by comparing the sample with the standard solution of caffeine. The results indicated that the caffeine concentration deduced from fluorescence measurement was strongly linked to the value obtained by HPLC (Figure 4), which showed the accuracy and effectiveness of the experimental method.

## 4. Conclusions

In this work, we design a rapid, high-selectivity and -sensitivity fluorescence method to determine caffeine content in actual tea infusion and tea beverages using 3,5-diaminobenzoic acid as a probe. The noncovalent interaction between caffeine and 3,5-diaminobenzoic acid are investigated by molecular electrostatic potential analysis and fluorescent lifetime. In addition, the optimum conditions were found by fluorescence experiment. Under the optimum conditions, a linear relation was found in the range of 0.1–100 μM and the limit of detection of caffeine was 0.03 μM. This method has been applied to the quantitative analysis of caffeine in commercial tea beverages and compared with the results of HPLC. The results show that the method is effective. In summary, this method was efficient, low cost and environmentally friendly in comparison with traditional method. This shows that this method could be widely used to determine caffeine in tea infusion and tea beverages.

## Figures and Tables

**Table 1:** Different methods for the determination of caffeine.

Detection Method	Linear Range (μM)	Limit of Detection (μM)	Ref.
UV–vis	12.5–142 μM	8.5 μM	[29]
amperometric detection	0.83–300 μM	0.4 μM	[30]
square wave voltammetry	0.5–100 μM	0.1 μM	[31]
HPLC	8.8–114 μM	11.6 μM	[7]
GC–MS	0.25–25 μM	0.02 μM	[8]
fluorescence	0.1–100 μM	0.03 μM	This work

**Table 2:** Detection of caffeine in actual samples (n = 3).

Sample	Added (μM)	Found (μM, n = 3) ^a^	Recovery (%, n = 3)
S1	0	8.70	103.8
	5	13.88	
S2	0	12.05	96.0
	5	16.85	
S3	0	6.87	103.0
	5	12.02	
S4	0	4.10	97.4
	5	8.97	
S5	0	5.39	96.6
	5	10.22	
S6	0	3.53	106.2
	5	8.84	

^a^ n = 3 indicates that all experimental data are averaged after three experiments.

## Supplementary Materials

The following are available online at https://www.mdpi.com/1424-8220/20/3/819/s1, Figure S1: Time-resolved fluorescence decay of 3,5-diaminobenzoic acid (probe) and caffeine with 3,5-diaminobenzoic acid (probe). Figure S2: (a) Effects of concentration of 3,5-diaminobenzoic acid on fluorescence intensity. a. Percentage of fluorescence quenching at different concentrations; b. Normalized fluorescence intensity. (b) Effects of pH on fluorescence-quenching intensity. Figure S3: The sigmoidal fitting of pH with fluorescence intensity. Figure S4: (a) Effects of temperature on fluorescence-quenching intensity. (b) Effects of time on fluorescence-quenching intensity. Figure S5: HPLC for the caffeine, the peak at 1 represents caffeine. Figure S6: HPLC for the tea infusion S1, the peak at 1 represents caffeine. Figure S7: HPLC for the tea infusion S2, the peak at 1 represents caffeine. Figure S8: HPLC for the tea infusion S3, the peak at 1 represents caffeine. Figure S9: HPLC for the tea infusion S4, the peak at 1 represents caffeine. Figure S10: HPLC for the tea infusion S5, the peak at 1 represents caffeine. Figure S11: HPLC for the tea infusion S6, the peak at 1 represents caffeine.
