# Supplementary material for: Fluorescence Sensing of Caffeine in Tea Beverages with 3,5-diaminobenzoic Acid
Source: Sensors (Basel). 2020 Feb 3;20(3):819. doi: 10.3390/s20030819 (PMC7038766; doi:10.3390/s20030819)
Supplement: Supplementary file 1 [file sensors-20-00819-s001.pdf]

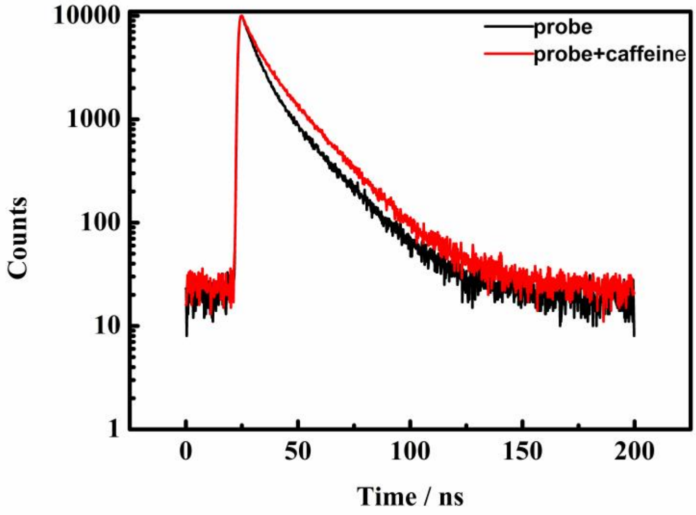

Figure S1. Time-resolved fluorescence decay of 3,5-diaminobenzoic acid (probe) and caffeine with 3,5-diaminobenzoic acid (probe).

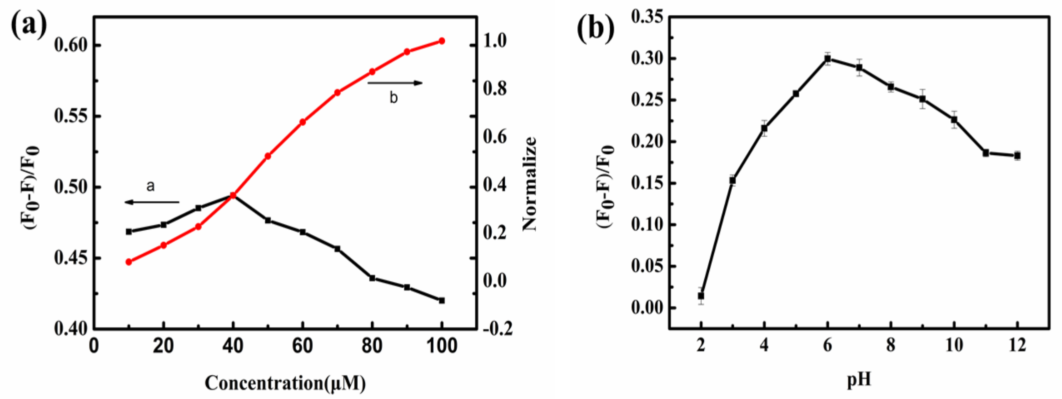

Figure S2. (a) Effects of concentration of 3,5-diaminobenzoic acid on fluorescence intensity. a. Percentage of fluorescence quenching at different concentrations; b. Normalized fluorescence intensity. (b) Effects of pH on fluorescence quenching intensity.

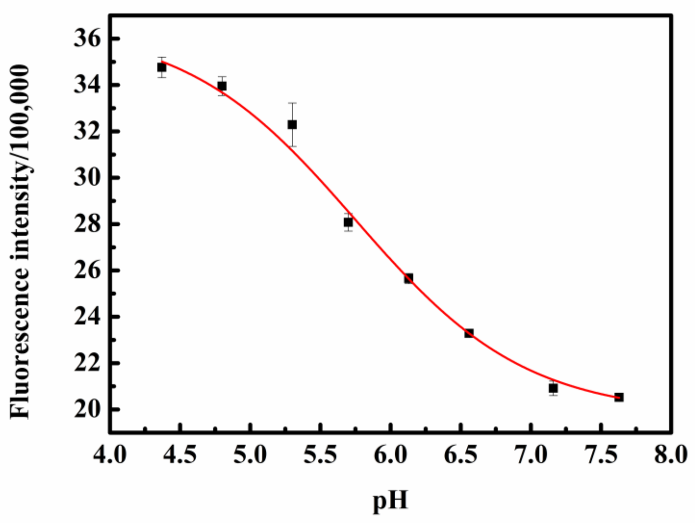

Figure S3. The sigmoidal fitting of pH with fluorescence intensity

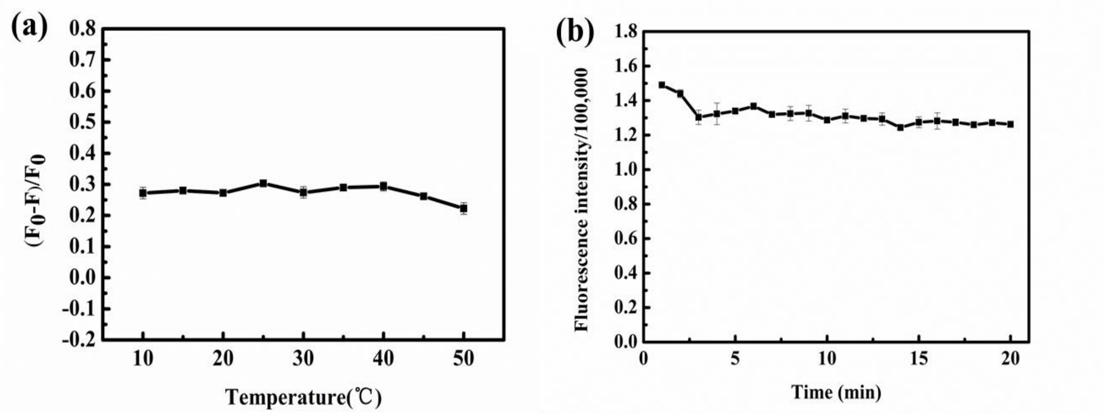

Figure S4. (a) Effects of temperature on fluorescence quenching intensity. (b) Effects of time on fluorescence quenching intensity.

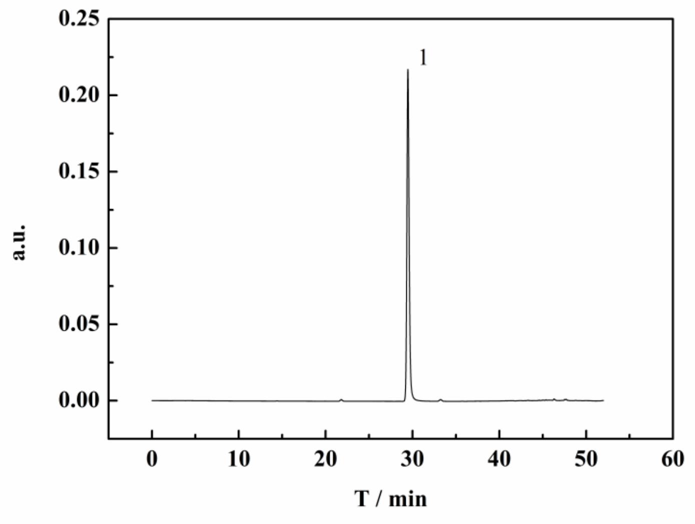

Figure S5. HPLC for the caffeine, the peak at 1 represents caffeine

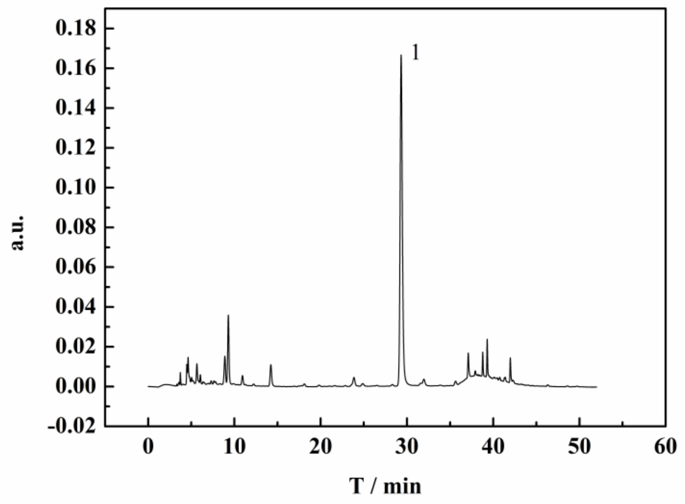

Figure S6. HPLC for the tea infusion S1, the peak at 1 represents caffeine

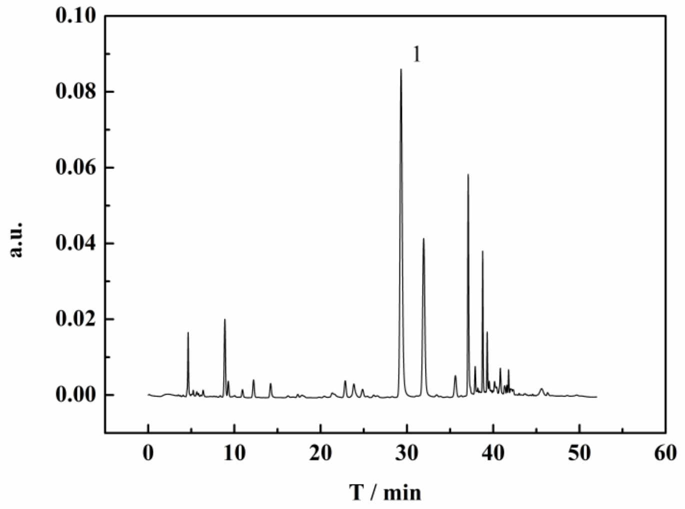

Figure S7. HPLC for the tea infusion S2, the peak at 1 represents caffeine

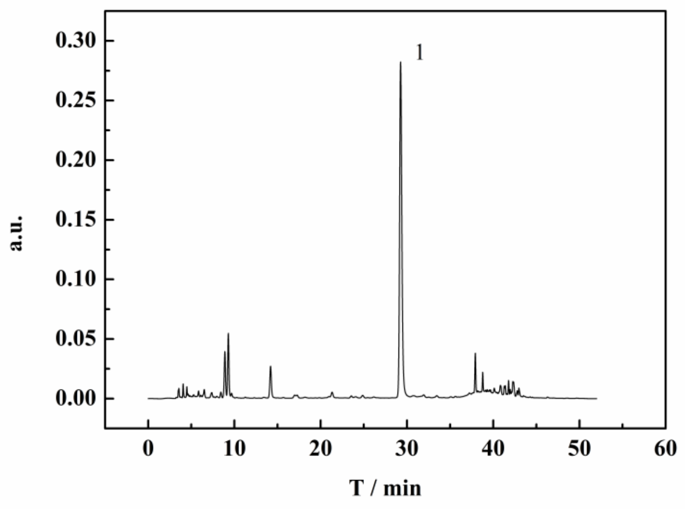

Figure S8. HPLC for the tea infusion S3, the peak at 1 represents caffeine

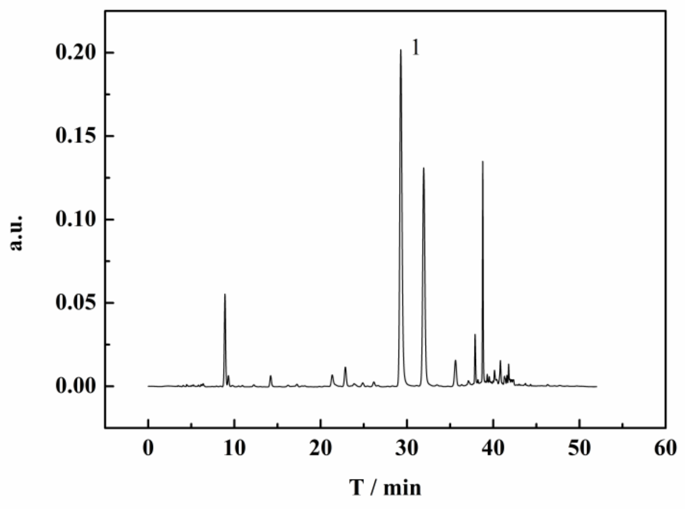

Figure S9. HPLC for the tea infusion S4, the peak at 1 represents caffeine

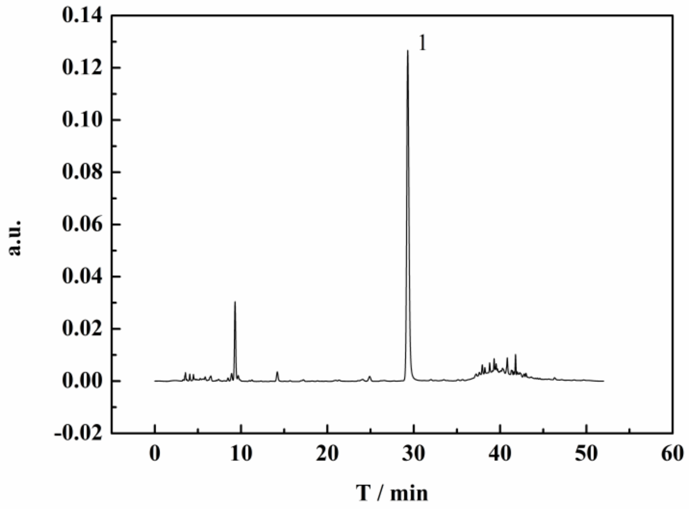

Figure S10. HPLC for the tea infusion S5, the peak at 1 represents caffeine

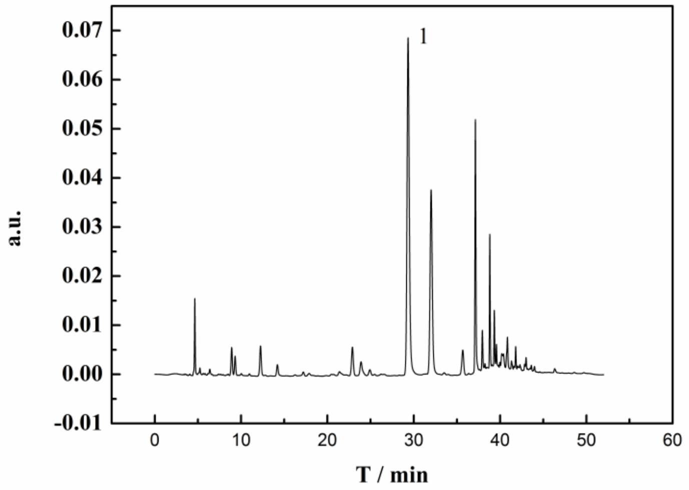

Figure S11. HPLC for the tea infusion S6, the peak at 1 represents caffeine
